# The relationship between perceived organizational support and psychological empowerment among nurses in selected South African public hospitals

**DOI:** 10.3389/fpsyg.2026.1859948

**Published:** 2026-08-26

**Authors:** L. L. Ratau

**Affiliations:** Department of Human Resource Management, Vaal University of Technology, Vanderbijlpark, South Africa

**Keywords:** employee empowerment, nurses, perceived organizational support, psychological empowerment, public hospitals

## Abstract

**Orientation:**

Psychological empowerment is essential for improving employee effectiveness in demanding work environments such as public healthcare, where organizational support plays an important role in shaping employees’ work experiences.

**Research purpose:**

This study examined the relationship between perceived organizational support and psychological empowerment among nurses in the public healthcare sector.

**Motivation for the study:**

South African public healthcare institutions face high workloads and resource constraints, highlighting the need to understand how organizational support improves employees’ psychological functioning.

**Research approach/design and method:**

A quantitative cross-sectional survey design was employed. Data were collected from 230 nurses using a structured questionnaire and analysed using the SPSS Version 27.

**Main findings:**

The results indicated a significant positive relationship between perceived organizational support and psychological empowerment, indicating that higher organizational support is associated with greater empowerment among nurses.

**Practical/managerial implications:**

The results suggest that healthcare management should prioritize supportive organizational practices, such as recognition, communication, and professional development, to improve employees’ sense of autonomy, competence, and impact.

**Contribution/value-add:**

This study contributes to the literature by highlighting the importance of perceived organizational support as a key driver of psychological empowerment within the South African public healthcare context.

## Introduction

Access to healthcare is recognized as a fundamental human right under Section 27 of the Constitution of the Republic of South Africa (1996), which guarantees everyone the right to have access to healthcare services and obliges the State to take reasonable legislative and other measures to progressively realize this right. Despite this constitutional commitment, South Africa’s public healthcare system continues to experience significant challenges that compromise the effective delivery of equitable and quality healthcare services (Maphumulo and Bhengu, 2019). Public healthcare systems, particularly in developing countries such as South Africa, are under increasing pressure to deliver high-quality healthcare amid resource constraints, staff shortages and rising patient demands (Abrahams et al., 2022; Ngobeni et al., 2020). These challenges are further compounded by structural inequalities, limited healthcare funding, infrastructure constraints and the increasing burden of communicable and non-communicable diseases (Pamu et al., 2025). In addition, Mkhwanazi et al. (2026) state that the aftermath of the COVID-19 pandemic has intensified existing pressures, placing further strain on already fragile healthcare systems. Consequently, public healthcare institutions are required to deliver efficient and quality care despite operating within increasingly constrained organizational environments (Beheshtinia et al., 2025). According to the National Department of Health (2025), persistent workforce shortages, delays in filling funded healthcare posts, and shortages of critical nurses continue to impede equitable healthcare delivery in South Africa. These human resource deficits constrain the health system’s capacity to provide accessible, high-quality healthcare services and remain a major challenge to strengthening public health service delivery (Malakoane et al., 2020). Similarly, the National Department of Health (2020), through the *2030 Human Resources for Health Strateg*y acknowledges that South Africa faces substantial shortages and inequitable distribution of healthcare professionals across provinces and between the public and private sectors. The consequences of these systemic challenges extend beyond organizational performance and have significant implications for both healthcare professionals and patients (Krijgsheld et al., 2022).

Healthcare professionals, especially nurses, remain central to healthcare service delivery and are often required to perform their duties under conditions characterized by excessive workloads, emotional strain and organizational complexity (Cranage and Foster, 2022). Huhtala et al. (2021) and Al Fayez et al. (2024) argue that these demanding working conditions not only compromise employees’ psychological well-being but also influence their motivation, job performance and ultimately the quality of patient care. Similarly, Ooms et al. (2022) note that prolonged exposure to occupational stress and increasing workload demands may result in burnout, reduced job satisfaction and declining organizational effectiveness. Syahrir and Falah (2021) further contend that these challenging conditions necessitate organizational interventions that strengthen employees’ psychological functioning and workplace effectiveness. Beyond their effects on employees, these challenges may also contribute to increased medical errors, longer waiting times, declining patient satisfaction and escalating medico-legal claims against provincial Departments of Health (Hostiuc and Gherghiceanu, 2026; Ndebele, 2024). According to the Portfolio Committee on Health (2024), medico-legal claims continue to impose a substantial financial burden on South Africa’s public healthcare system. The committee reported that the nine provincial health departments paid approximately R1.5 billion in medico-legal claims during the 2023/24 financial year, while outstanding contingent liabilities amounted to R63.5 billion, posing a significant risk to the sustainability of the public health system and diverting resources away from essential healthcare services. Likewise, the Parliamentary Budget Office (2022) reported that the average nurse-to-patient ratio in South Africa’s public healthcare sector is approximately 1:218, compared with an ideal ratio of 1:16, further illustrating the significant workload pressures experienced by nurses and the increasing strain on healthcare professionals. Consequently, there is growing recognition that improving healthcare outcomes requires not only structural and operational reforms but also organizational strategies that strengthen employees’ psychological resources and workplace experiences (Brunetto et al., 2020). Supporting this view, Søvold et al. (2021) argue that improving nurses’ psychological functioning is essential for sustaining workforce performance and maintaining effective healthcare delivery.

Against this backdrop, increasing scholarly attention has been directed toward psychological empowerment as a strategic organizational resource capable of improving employee effectiveness within demanding healthcare environments (Şenol Çelik et al., 2024). Psychological empowerment reflects an individual’s intrinsic motivation and active engagement with work roles (Al Otaibi et al., 2023). According to Al Otaibi et al. (2023), psychologically empowered nurses are more likely to feel competent, autonomous and capable of making meaningful contributions to organizational objectives, thereby improving both individual and organizational outcomes. In healthcare settings, where rapid decision-making, professional judgement and effective teamwork are essential, psychological empowerment becomes an important determinant of employee effectiveness and quality patient care (Alharbi et al., 2025). Previous research has consistently demonstrated that psychologically empowered employees report higher levels of work engagement, innovation, organizational commitment and job satisfaction while experiencing lower levels of burnout and emotional exhaustion (Al Otaibi et al., 2023; Orłowska and Laguna, 2023). However, although research on psychological empowerment among nurses in South Africa remains limited, studies by Chikobvu and Harunavamwe (2022), and Thobane et al. (2022) have highlighted the importance of strengthening healthcare workers’ psychological well-being, resilience, work engagement, and psychosocial support to improve employee performance and the quality of patient care. Although psychological empowerment is recognized as an important organizational resource, it does not develop in isolation but is influenced by the organizational context within which employees perform their work (Llorente-Alonso et al., 2024). Perceived organizational support (POS), defined as employees’ belief that their organization values their contributions and cares about their well-being, has emerged as a key antecedent of positive employee attitudes and psychological functioning (Eisenberger et al., 2020). Within healthcare settings characterized by high workloads, emotional demands and resource constraints, organizational support serves as an important psychological resource that enables healthcare professionals to cope with workplace challenges while maintaining motivation and commitment (Moustafa et al., 2024). Employees who perceive adequate organizational support through fair practices, effective communication, recognition, supervisor support, and access to resources are more likely to develop positive work attitudes and experience greater well-being, work engagement, resilience, and reduced burnout (Moustafa et al., 2024). In public healthcare settings, perceived organizational support may strengthen nurses’ psychological resources and enhance the quality of patient care. However, despite growing international evidence, limited research has examined the relationship between perceived organizational support and psychological empowerment within South African public healthcare institutions (Chikobvu and Harunavamwe, 2022; Ratau, 2025; Thobane et al., 2022).

The present study is underpinned by Organizational Support Theory (Eisenberger et al., 1986) and Psychological Empowerment Theory (Spreitzer, 1995), which together provide a theoretical explanation of the relationship examined in this study. Organizational Support Theory developed by Eisenberger et al. (1986) proposes that employees develop global beliefs regarding the extent to which their organization values their contributions and cares about their well-being. Eisenberger et al. (1986) states that when employees perceive high levels of organizational support, they are more likely to reciprocate through positive attitudes and behaviors because they believe their organization recognizes their efforts and values their welfare. Complementing this perspective, Psychological Empowerment Theory developed by Spreitzer (1995), explains how supportive organizational environments enhance employees’ intrinsic motivation by strengthening their perceptions of meaning, competence, self-determination and impact in the workplace (Spreitzer, 1995). Organizational Support Theory explains why supportive organizational practices influence employees’ psychological experiences (Eisenberger et al., 1986), whereas Psychological Empowerment Theory explains how these supportive organizational conditions translate into greater intrinsic motivation and psychological empowerment (Spreitzer, 1995). These theories provide a conceptual framework for understanding how organizational support may strengthen psychological empowerment among nurses working within resource-constrained public healthcare environments. Although the importance of perceived organizational support and psychological empowerment has been widely recognized in organizational behavior literature, important gaps remain within the South African public healthcare context. Previous international studies have predominantly focused on examining the relationships between perceived organizational support and outcomes such as job satisfaction, organizational commitment, employee engagement, burnout and turnover intention (Rafiq et al., 2020; Gu et al., 2022; Xue et al., 2022; Ding and Wu, 2023). Existing South African research has similarly focused on employee outcomes such as burnout, retention, organizational commitment and job satisfaction (Ditlopo et al., 2024; Lerotholi and Bezuidenhout, 2023; Thobane et al., 2022), leaving limited empirical evidence regarding the relationship between perceived organizational support and psychological empowerment among nurses. Furthermore, organizational and healthcare system differences between developed countries and South Africa limit the direct transferability of existing international findings to the South African public healthcare context. Consequently, there remains a need for context-specific empirical evidence that explains how organizational support contributes to nurses’ psychological empowerment within South African public hospitals. Addressing this gap is particularly important because psychologically empowered nurses are better equipped to remain motivated, resilient and effective despite challenging organizational conditions, thereby contributing to improved healthcare service delivery and patient outcomes (Moloney et al., 2020). Accordingly, this study examines the relationship between perceived organizational support and psychological empowerment among nurses employed in selected South African public hospitals.

## Literature review

### Perceived organizational support

Perceived organizational support (POS) is a well-established construct in organizational behavior that reflects employees’ general belief that their organization values their contributions and genuinely cares about their well-being (Pham et al., 2024). The construct originates from Organizational Support Theory developed by Eisenberger et al. (1986), which proposes that employees develop global perceptions regarding the extent to which their organization appreciates their efforts and is concerned about their welfare. These perceptions are formed through employees’ day-to-day interactions with organizational policies, managerial practices and workplace experiences. According to Organizational Support Theory, when employees perceive that they are valued and supported, they are more likely to reciprocate through positive attitudes, stronger organizational commitment and behaviors that contribute to organizational effectiveness (Eisenberger et al., 1986).

Organizational support is communicated through both formal organizational practices and informal interpersonal interactions (Eğriboyun, 2022). Formal mechanisms of organizational support include fair policies, equitable reward systems, professional development opportunities, adequate resources, and transparent decision-making, while informal mechanisms encompass supervisor support, recognition, respect, constructive feedback, and emotional encouragement (Pham et al., 2024). These organizational experiences shape employees’ perceptions regarding the extent to which the organization values their contribution and supports their professional and personal well-being (Eisenberger et al., 2020). Within healthcare settings, perceived organizational support assumes even greater importance because nurses routinely operate in environments characterized by heavy workloads, emotional labor, staff shortages and limited organizational resources (Alnasser, 2026). Under such conditions, supportive organizational practices such as adequate staffing, access to medical equipment, participative leadership, recognition and emotional support become essential organizational resources that enable nurses to cope with demanding work conditions (Bharathi and Sujatha, 2024; Pattali et al., 2024). These supportive practices not only assist employees in performing their clinical responsibilities effectively but also reinforce their confidence that the organization values their contribution despite the challenges associated with public healthcare delivery. Within the South African public healthcare sector, these organizational resources are particularly important given the persistent workforce shortages, resource constraints and increasing patient demands (Ditlopo et al., 2024; Lerotholi and Bezuidenhout, 2023; National Department of Health, 2025; Thobane et al., 2022).

Empirical evidence consistently demonstrates that perceived organizational support is associated with numerous favorable employee and organizational outcomes. For example, employees who perceive high levels of organizational support generally report greater job satisfaction, stronger organizational commitment, higher levels of work engagement and improved psychological well-being (Putranto et al., 2021; Pelealu, 2022; Duong et al., 2024). Similarly, Moustafa et al. (2024) argue that organizational support serves as a valuable psychological resource that reduces burnout by strengthening employees’ psychological capital and resilience. Within healthcare environments, where employees frequently experience occupational stress, emotional exhaustion and uncertainty, organizational support provides an important protective mechanism that enables employees to cope effectively with workplace demands (K’osuri et al., 2020). Although the beneficial effects of perceived organizational support have been extensively documented, previous studies have largely concentrated on outcomes such as job satisfaction, organizational commitment, employee engagement, turnover intention and psychological well-being (Putranto et al., 2021; Pelealu, 2022; Duong et al., 2024). Comparatively fewer studies have examined perceived organizational support as an antecedent of psychological empowerment, particularly within resource-constrained public healthcare environments.

### Psychological empowerment

Psychological empowerment is widely recognized as an intrinsic motivational construct that reflects employees’ active orientation toward their work roles and their belief that they possess the capability, autonomy and influence necessary to perform their responsibilities effectively (Oliveira et al., 2023). Unlike structural empowerment, which focuses on organizational policies and access to resources, psychological empowerment emphasizes employees’ cognitive appraisal of their work experiences and the extent to which they perceive themselves as capable of influencing organizational outcomes (Spreitzer, 1995). According to Psychological Empowerment Theory by Spreitzer (1995), psychological empowerment comprises four interrelated cognitive dimensions namely, meaning, competence, self-determination and impact, which shape how employees perceive their work roles and responsibilities within the organization. Meaning refers to the extent to which employees perceive their work as personally valuable, competence reflects their confidence in performing work-related tasks effectively, self-determination relates to employees’ sense of autonomy in regulating work activities, while impact refers to their perceived ability to influence organizational outcomes (Spreitzer, 1995). Although conceptually distinct, these four dimensions interact to shape employees’ overall experience of psychological empowerment and their willingness to engage proactively in their work (Rani et al., 2021). Within healthcare settings, psychological empowerment assumes particular importance because nurses are required to make timely clinical decisions, respond to rapidly changing patient conditions and collaborate effectively within multidisciplinary teams.

Employees who perceive their work as meaningful, feel competent in their clinical abilities, exercise professional autonomy and believe they can positively influence patient outcomes are generally better equipped to deliver high-quality healthcare services (Al Otaibi et al., 2023). In contrast, nurses who experience low levels of empowerment may become less confident in their decision-making, less engaged in patient care and more susceptible to occupational stress and emotional exhaustion (Al-Shomrani et al., 2024). Given the complexity of healthcare environments, psychological empowerment therefore represents an important mechanism through which healthcare organizations can enhance both employee effectiveness and patient care outcomes. Previous studies have associated psychological empowerment with improved job performance, greater innovation, stronger organizational commitment, higher job satisfaction and reduced burnout (Orłowska and Laguna, 2023; Al-Shomrani et al., 2024). Furthermore, Llorente-Alonso et al. (2024), in their meta-analysis, identified psychological empowerment as a key mechanism linking supportive organizational environments to desirable work attitudes and behaviors across diverse organizational settings. These findings suggest that psychologically empowered employees are more resilient, adaptable and motivated to contribute beyond their prescribed job responsibilities, particularly in occupations characterized by high levels of responsibility and uncertainty. Despite substantial evidence supporting the benefits of psychological empowerment, several gaps remain within the existing literature. The current research has predominantly focused on the consequences of psychological empowerment in private-sector organizations and healthcare systems in developed countries, with limited attention to the organizational conditions that facilitate psychological empowerment in public healthcare settings, thereby limiting the applicability of existing evidence to South Africa’s public hospitals (Gathmyr et al., 2024; Llorente-Alonso et al., 2024). Considering the persistent workforce shortages, resource constraints and demanding working conditions that characterize South African public hospitals, understanding the antecedents of psychological empowerment within this context remains an important area for empirical investigation.

### Perceived organizational support and psychological empowerment

Perceived organizational support has increasingly been recognized as an important organizational antecedent of psychological empowerment because it provides employees with the social, emotional and instrumental resources necessary to perform their work effectively (Kumar et al., 2022; Park and Kim, 2022). Organizational Support Theory by Eisenberger et al. (1986) proposes that when employees perceive that their organization values their contributions, recognizes their efforts and genuinely cares about their well-being, they develop positive psychological responses that strengthen their relationship with the organization. Within healthcare settings, where professionals routinely encounter high workloads, emotional demands and resource limitations, supportive organizational practices can therefore create conditions that enhance employees’ psychological empowerment (Orłowska and Laguna, 2023).

From a theoretical perspective, perceived organizational support contributes to each dimension of psychological empowerment proposed by Spreitzer (1995). Employees who receive recognition and organizational appreciation are more likely to perceive their work as meaningful because they understand how their contributions support broader organizational objectives (Govender and Bussin, 2020). Similarly, organizations that provide adequate training, mentoring, performance feedback and access to resources strengthen employees’ confidence in their professional capabilities, thereby enhancing their sense of competence (Spreitzer, 1995). Participative management practices and opportunities for shared decision-making promote employees’ autonomy, contributing to greater self-determination, while organizational trust and supportive leadership increase employees’ perceptions that they can influence organizational decisions and patient care outcomes, thereby strengthening their sense of impact (Spreitzer, 1995). Consequently, supportive organizational environments not only improve employees’ perceptions of organizational care but also create favorable psychological conditions through which empowerment can develop (Llorente-Alonso et al., 2024).

**FIGURE 1:**
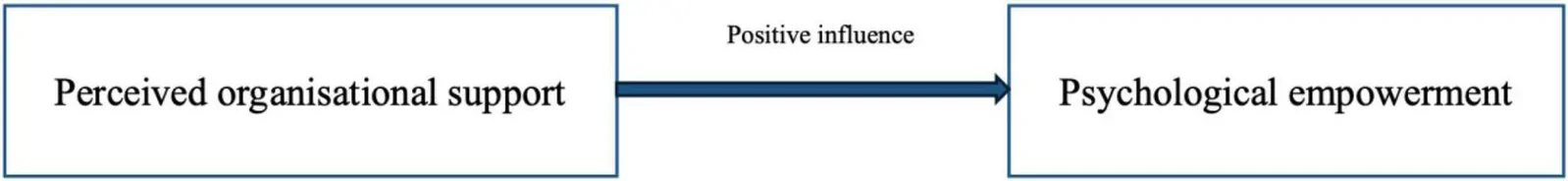
Research model.

Previous research supports the positive relationship between perceived organizational support and psychological empowerment across different organizational settings. For example, Kumar et al. (2022) found that employees who perceived higher organizational support reported greater levels of empowerment and were more willing to engage in proactive workplace behaviors. Similarly, Maan et al. (2020) demonstrated that psychological empowerment partially explained how perceived organizational support contributed to improved employee attitudes and job satisfaction, suggesting that organizational support enhances employee outcomes by strengthening positive psychological states. K’osuri et al. (2020) further argue that within healthcare organizations, organizational support serves as an essential psychological resource that promotes empowerment and employee engagement, particularly in demanding public hospitals. These previous studies indicate that organizational support extends beyond improving employee satisfaction and commitment; it also strengthens employees’ intrinsic motivation and their confidence in performing their professional responsibilities effectively. Building on Organizational Support Theory (Eisenberger et al., 1986), Psychological Empowerment Theory (Spreitzer, 1995) and the existing empirical evidence, this study proposes that employees who perceive higher levels of organizational support are more likely to experience greater psychological empowerment. Thus, it is hypothesized that perceived organizational support is positively associated with psychological empowerment among nurses in selected South African public hospitals, as illustrated in Figure 1.

## Research methodology

### Research design

#### Research approach

; This study adopted a quantitative research approach using a cross-sectional survey design to examine the relationship between perceived organizational support and psychological empowerment among nurses employed in selected public hospitals in South Africa. A quantitative approach was considered appropriate because the study sought to objectively measure the relationship between predefined variables using numerical data and statistical techniques. Quantitative research enables the testing of hypotheses and facilitates the generalization of findings from a representative sample to the target population (Creswell and Creswell, 2017). A cross-sectional survey design was employed because data were collected from participants at a single point in time. This design is appropriate for examining relationships between variables as they naturally occur without manipulating the research environment (Sanders et al., 2019).

#### Research method

The study employed the survey research method to collect quantitative data from nurses employed at Tshilidzini Hospital and Siloam Hospital in the Vhembe District of Limpopo Province. A paper-based, self-administered questionnaire was used to obtain responses from participants during the data collection period. The survey method was considered appropriate because it enabled data to be collected efficiently from a relatively large number of participants.

#### Research setting

The study was conducted in the Vhembe District Municipality, Limpopo Province, South Africa. The district was considered an appropriate setting for examining organizational support and psychological empowerment among nurses working within public hospitals. Two public hospitals were selected for the study, namely Tshilidzini Hospital and Siloam Hospital. Tshilidzini Hospital was selected because it is the largest and one of the oldest referral hospitals in the Vhembe District, serving a large patient population and employing a substantial nursing workforce. Siloam Hospital was selected because it is the newest public hospital within the district, providing an opportunity to include nurses working in a more recently established healthcare institution. Including these two hospitals allowed the study to capture perceptions from nurses working in different organizational contexts within the same district.

#### Research respondents and sampling

The target population comprised approximately 850 nurses employed at Tshilidzini Hospital and Siloam Hospital in the Vhembe District, Limpopo Province. This population figure was obtained from workforce records published by the Limpopo Provincial Department of Health. Nurses were selected as the study population because they constitute the largest professional group within public hospitals and play a critical role in delivering healthcare services under often demanding organizational conditions. The required sample size was determined using the Raosoft Sample Size Calculator, based on a population of approximately 850 nurses, a 95% confidence level, a 5% margin of error, and a 50% response distribution. The calculator recommended a minimum sample size of 206 participants. To account for possible incomplete or unusable questionnaires, 230 paper-based questionnaires were distributed. A stratified convenience sampling technique was employed to recruit participants. The target population was first divided into two strata according to the participating hospitals, with Siloam Hospital constituting the first stratum and Tshilidzini Hospital the second stratum. Proportional allocation was used to distribute the questionnaires across the two hospitals, with 110 questionnaires distributed at Siloam Hospital and 120 questionnaires distributed at Tshilidzini Hospital. Within each hospital, nurses who met the inclusion criteria and were available and willing to participate during the data collection period were recruited using convenience sampling. All 230 completed questionnaires were suitable for analysis. The demographic characteristics of the respondents are presented in Table 1.

**TABLE 1:** Demographic information of nurses.

Variable	*n* (%)
Gender	
Male	86 (37%)
Female	144 (63%)
Age	
20–25 yrs	16 (7%)
26–30 yrs	33 (14%)
31–40 yrs	50 (22%)
41–50 yrs	80 (35%)
51+ yrs	51 (22%)
Highest qualification	
Certificate	44 (19%)
Diploma	79 (34%)
Degree	89 (39%)
Postgraduate	16 (7%)
Other	2 (1%)
Length of service	
0–5 yrs	46 (20%)
6–10 yrs	79 (34%)
11–15 yrs	58 (25%)
16+ yrs	47 (21%)
Rank/Designation	
Nursing assistant	66 (29%)
LPN	22 (10%)
Registered nurse	111 (48%)
APRN	22 (10%)
DNP	9 (4%)
Job status	
Permanent	230 (100%)
Contract	0 (0%)

LPN, licensed practical nurse; APRN, advanced practice registered nurse; DNP, Doctor of Nursing Practice.

### Measuring instruments

Data were collected using a structured questionnaire comprising 22 items adapted from two well-established and validated measurement instruments. Perceived organizational support was measured using the 10-item Survey of Perceived Organizational Support developed by Eisenberger et al. (1986), with Cronbach’s alpha coefficients ranging between 0.88 and 0.91. Psychological empowerment was measured using the 12-item Psychological Empowerment Scale developed by Spreitzer (1995). The instrument measures four dimensions of psychological empowerment, namely meaning, competence, self-determination, and impact. Previous studies have reported Cronbach’s alpha coefficients ranging between 0.82 and 0.89, demonstrating satisfactory reliability across organizational and healthcare settings. All questionnaire items were measured using a five-point Likert scale, ranging from 1 (strongly disagree) to 5 (strongly agree). The internal consistency of both instruments was reassessed using Cronbach’s alpha coefficients. Items that negatively affected reliability were removed based on the results of the reliability analysis, consistent with the recommendations of Nunnally (1978).

### Research procedure

Paper-based questionnaires were distributed to eligible nurses during scheduled periods agreed upon with hospital management to minimize disruption to clinical activities. Participants received an information letter explaining the purpose of the study, the voluntary nature of participation, and their rights as research participants. Those who agreed to participate provided written informed consent before completing the questionnaire independently. Completed questionnaires were placed in sealed collection boxes located in the nursing managers’ offices, thereby ensuring confidentiality throughout the data collection process.

### Ethical considerations

Ethical approval for the study was obtained from the University of Venda Research Ethics Committee (Reference No. SMS/19/HRM/01/0409) before data collection commenced. Permission to conduct the study was first obtained from the Limpopo Provincial Department of Health. In accordance with the Department’s research governance procedures, approval was subsequently granted by the Vhembe District Department of Health before permission was secured from the management of the participating hospitals, namely Tshilidzini Hospital and Siloam Hospital. Data collection commenced only after all the required ethical and institutional approvals had been obtained. Participation was entirely voluntary, and respondents were informed of the purpose of the study and their right to withdraw from the study at any stage without penalty. Written informed consent was obtained from all participants before they completed the questionnaire. Participants were assured that their responses would remain anonymous and confidential, that no personally identifying information would be collected, and that all data would be securely stored and used exclusively for research purposes.

### Statistical analysis

Data were analysed using IBM SPSS Statistics Version 27. Descriptive statistics, including frequencies, percentages, means, standard deviations, skewness, and kurtosis, were computed to summarize respondents’ demographic characteristics and examine the distribution of the study variables. Exploratory factor analysis (EFA) was conducted to evaluate the underlying factor structure of the measurement instruments and confirm their suitability within the South African public healthcare context. Reliability analysis was performed using Cronbach’s alpha coefficients, with values of 0.70 or above regarded as indicative of acceptable internal consistency (Taber, 2018). Pearson’s product-moment correlation analysis was conducted to examine the relationship between perceived organizational support and psychological empowerment. Thereafter, simple linear regression analysis was performed to determine the extent to which perceived organizational support predicted psychological empowerment among nurses. All statistical analyses were conducted using a 95% confidence level (α = 0.05), which is widely accepted in behavioral and health sciences as providing an appropriate balance between Type I and Type II error rates (Field, 2018).

## Results

### Reliability analysis

Item analysis and reliability testing were conducted to ensure internal consistency and suitability for further analysis. Cronbach’s alpha values above 0.70 were considered acceptable (Taber, 2018). The psychological empowerment scale demonstrated adequate reliability, consistent with Spreitzer’s (1995) multidimensional conceptualization. The perceived organizational support scale also achieved acceptable reliability after the removal of poorly performing items, supporting its use in assessing employees’ perceptions of organizational care and valuation (Eisenberger et al., 1986). Table 2 presents the results of the reliability analysis.

**TABLE 2:** Summary of reliability analysis.

Scale	Cronbach’s α	Decision
Psychological empowerment (PE)	0.739	Adequate
Perceived organisational support (POS)	0.711	Adequate

### Exploratory factor analysis

Exploratory factor analysis confirmed the suitability and dimensionality of the measurement instruments. Both perceived organizational support and psychological empowerment demonstrated acceptable sampling adequacy and significant Bartlett’s test results, indicating that the data were appropriate for factor analysis. The results supported the multidimensional nature of psychological empowerment and the dual nature of organizational support (socio-emotional and instrumental), consistent with existing literature (Samuel and Engelbrecht, 2021). The EFA results are presented in Table 3.

**TABLE 3:** EFA results.

Construct	KMO	Bartlett’s χ^2^	df	*p*	Number of factors	Variance explained
Perceived organizational support	0.763	225.887	21	< 0.001	2	53.16%
Psychological empowerment	0.730	568.741	66	< 0.001	2	35.74%

### Descriptive statistics

Descriptive statistics were computed to examine the central tendencies and distribution of the data. As shown in Table 4, respondents reported generally favorable perceptions of both perceived organizational support and psychological empowerment. Perceived organizational support recorded a mean score of 3.6 (SD = 4.37), while psychological empowerment yielded a slightly higher mean of 3.9 (SD = 4.66), indicating a relatively positive evaluation of both constructs among nurses. Furthermore, the skewness and kurtosis values presented in Table 4 indicate that the data were approximately normally distributed. Specifically, perceived organizational support had a skewness of −0.075 and kurtosis of −0.822, while psychological empowerment showed a skewness of −0.582 and kurtosis of −0.881. These values fall within acceptable ranges, suggesting no serious violations of normality and supporting the use of parametric statistical analyses.

**TABLE 4:** Descriptive statistics.

Construct	Mean	SD	Skewness	Kurtosis
Perceived organizational support	3.6	0.437	−0.075	−0.822
Psychological empowerment	3.9	0.466	−0.582	−0.881

### Correlation analysis

Pearson correlation analysis was conducted to examine the relationship between perceived organizational support and psychological empowerment. As shown in Table 5, the results indicate a statistically significant positive relationship between perceived organizational support and psychological empowerment (*r* = 0.331, *p* < 0.01). This result indicates that higher levels of perceived organizational support are associated with higher levels of psychological empowerment among nurses. This result is consistent with Organizational Support Theory (Eisenberger et al., 1986), which posits that when employees perceive that their organization values their contributions and cares about their well-being, they are more likely to develop positive work-related attitudes and psychological states. In this study, perceived organizational support appears to function as a critical resource that improves employees’ sense of value and recognition, which in turn, promotes psychological empowerment. Furthermore, the result aligns with psychological empowerment theory (Spreitzer, 1995), which suggests that supportive organizational environments improve employees’ intrinsic motivation and their sense of meaning, competence, self-determination, and impact. In the context of public healthcare, where employees face high job demands, organizational support may strengthen these dimensions of empowerment by providing the necessary resources, encouragement, and autonomy required for effective performance (Al Otaibi et al., 2023). Most importantly, this result provides empirical support for the study’s hypothesis that perceived organizational support is positively associated with psychological empowerment among nurses in public healthcare.

**TABLE 5:** Correlations analysis.

Variable	1	2
Perceived organizational support	1	
Psychological empowerment	0.331**	1

**Correlation is significant at the 0.01 level (2-tailed).

### Regression analysis

Regression analysis was conducted to examine the role of perceived organizational support in predicting psychological empowerment. As shown in Table 6, perceived organizational support was found to be a significant positive predictor of psychological empowerment (β = 0.353, *t* = 5.30, df = 228, *p* < 0.01). This result indicates that higher levels of perceived organizational support are associated with increased levels of psychological empowerment among nurses. This finding is consistent with Organizational Support Theory (Eisenberger et al., 1986), which suggests that when employees perceive that their organization values their contributions and cares about their well-being, they are more likely to develop positive psychological states. In this study, perceived organizational support appears to serve as an important organizational resource that improves employees’ sense of value and recognition, thereby promoting empowerment. The result also supports psychological empowerment theory (Spreitzer, 1995), which posits that supportive organizational environments strengthen employees’ intrinsic motivation and their sense of meaning, competence, self-determination, and impact. Within the public healthcare context, organizational support in the form of resources, recognition, and supportive management practices may therefore play a critical role in improving these dimensions of empowerment. The results highlight the important role of organizational support in promoting empowerment among nurses, reinforcing the importance of supportive management practices in public healthcare settings.

**TABLE 6:** Regression analysis results.

Predictor	Outcome	β	t	df	*p*
Perceived organizational support	Psychological empowerment	0.353	5.30	228	< 0.01

β, standardized regression coefficient; t, t-value; df, degrees of freedom; p, significance level.

## Discussion

### Outline of the results

The results indicate that respondents generally reported relatively high levels of both perceived organizational support (*M* = 3.60, SD = 0.44) and psychological empowerment (*M* = 3.90, SD = 0.47). The skewness and kurtosis values for both constructs fell within acceptable ranges, suggesting that the data approximated a normal distribution and were suitable for subsequent parametric analyses. Pearson’s correlation analysis indicated a positive and statistically significant relationship between perceived organizational support and psychological empowerment (*r* = 0.331, *p* < 0.01), indicating that higher levels of perceived organizational support were associated with higher levels of psychological empowerment among nurses in the selected public hospitals. Simple linear regression analysis further demonstrated that perceived organizational support significantly predicted psychological empowerment (β = 0.353, t(228) = 5.30, *p* < 0.01). These findings support the study hypothesis that perceived organizational support is positively associated with psychological empowerment among nurses working in the selected South African public hospitals.

### Practical implications

The results of this study highlight the important role of perceived organizational support in promoting psychological empowerment among nurses in the selected public hospitals. Hospital management should therefore prioritize the development of supportive work environments that reinforce employees’ perceptions of being valued and cared for. From a practical perspective, healthcare institutions should implement targeted strategies aimed at strengthening employees’ sense of meaning, competence, self-determination, and impact in their roles. Firstly, management can introduce formal recognition programmes, such as employee-of-the-month awards or performance-based incentives, to acknowledge and reward staff contributions. Secondly, public hospitals should invest in continuous professional development, including regular training workshops, mentorship programmes, and opportunities for career advancement, to enhance employees’ competence and confidence in their roles. Thirdly, hospitals should promote participative decision-making by involving nurses in policy formulation, clinical decisions, and operational planning. These initiatives can strengthen nurses’ psychological empowerment and ultimately contribute to improved quality of patient care.

### Limitations and recommendations

This study is limited to nurses in selected public hospitals in Limpopo Province in the Vhembe district, and access to some hospitals was restricted, which may have affected the representativeness of the sample and limits the generalizability of the results to other districts. Additionally, the cross-sectional research design only captures a single point in time, which prevents conclusions about the direction or causality of the relationship between perceived organizational support and psychological empowerment. Future research should consider employing longitudinal designs to better examine how perceived organizational support influences psychological empowerment over time. Expanding the sample to include a larger and more diverse population across multiple provinces would also strengthen the generalizability of the findings. Furthermore, future studies could investigate additional outcomes of psychological empowerment such as employee engagement, performance, or retention and explore potential mediators or moderators that may affect the relationship between organizational support and psychological empowerment, including leadership styles, organizational culture or workload.

## Conclusion

The results indicate that organizational support is a significant contributor to employees’ sense of empowerment, highlighting that supportive work environments improve employees’ feelings of autonomy, competence, meaning and impact. The results align with Organizational Support Theory (Eisenberger et al., 1986), and Psychological Empowerment theory (Spreitzer, 1995), emphasizing the importance of organizational practices that value and support employees. Nurses who perceive higher levels of support are more likely to experience enhanced psychological empowerment, which is particularly important in demanding healthcare environments. In the context of public healthcare, sustaining perceived organizational support can allow institutions to cultivate a motivated, confident, and psychologically empowered workforce capable of delivering high-quality care and supporting long-term organizational effectiveness.

## Data Availability

The raw data supporting the conclusions of this article will be made available by the author, without undue reservation.
